# Encapsulation of the HSP-90 Chaperone Inhibitor 17-AAG in Stable Liposome Allow Increasing the Therapeutic Index as Assessed, *in vitro*, on *Leishmania (L) amazonensis* Amastigotes-Hosted in Mouse CBA Macrophages

**DOI:** 10.3389/fcimb.2018.00303

**Published:** 2018-08-30

**Authors:** Antonio Luis de Oliveira Almeida Petersen, Thiers A. Campos, Diana Angélica dos Santos Dantas, Juliana de Souza Rebouças, Juliana Cruz da Silva, Juliana P. B. de Menezes, Fábio R. Formiga, Janaina V. de Melo, Giovanna Machado, Patrícia S. T. Veras

**Affiliations:** ^1^Laboratory of Parasite-Host Interaction and Epidemiology (LAIPHE), Gonçalo Moniz Institute—FIOCRUZ, Salvador, Brazil; ^2^Graduate Program in Biological Sciences, Center of Biological Sciences, Federal University of Pernambuco, Recife, Brazil; ^3^Laboratory of Electron Microscopy and Microanalysis (LAMM), Center of Strategical Technologies (CETENE), Recife, Brazil; ^4^Institute of Biological Sciences, University of Pernambuco (UPE), Recife, Brazil; ^5^Postgraduate Program in Applied Cellular and Molecular Biology, Institute of Biological Sciences, University of Pernambuco (UPE), Recife, Brazil; ^6^Laboratory of Vector-Borne Infectious Diseases (LEITV), Gonçalo Moniz Institute—FIOCRUZ, Salvador, Brazil; ^7^National Institute of Technology in Tropical Diseases-National Council for Scientific and Technological Development, Brasilia, Brazil

**Keywords:** 17-AAG, Tanespimicyn, 2-hydroxypropyl-β-cyclodextrin, liposome, drug delivery systems, leishmaniasis, chemotherapy, HSP-90

## Abstract

The current long-term treatment for leishmaniasis causes severe side effects and resistance in some cases. An evaluation of the anti-leishmanial potential of an HSP90-inhibitor, 17-allylamino-17-demethoxygeldanamycin (17-AAG), demonstrated its potent effect against *Leishmania* spp. *in vitro* and *in vivo*. We have previously shown that 17-AAG can kill *L. (L) amazonensis* promastigotes with an IC_50_ of 65 nM and intracellular amastigote at concentrations as low as 125 nM. As this compound presents low solubility and high toxicity in human clinical trials, we prepared an inclusion complex containing hydroxypropyl-β-cyclodextrin and 17-AAG (17-AAG:HPβCD) to improve its solubility. This complex was characterized by scanning electron microscopy, and X-ray diffraction. Liposomes-containing 17-AAG:HPβCD was prepared and evaluated for encapsulation efficiency (EE%), particle size, polydispersity index (PDI), pH, and zeta potential, before and after accelerated and long-term stability testing. An evaluation of leishmanicidal activity against promastigotes and intracellular amastigotes of *L. (L) amazonensis* was also performed. The characterization techniques utilized confirmed the formation of the inclusion complex, HPβCD:17-AAG, with a resulting 33-fold-enhancement in compound water solubility. Stability studies revealed that 17-AAG:HPβCD-loaded liposomes were smaller than 200 nm, with 99% EE. Stability testing detected no alterations in PDI that was 0.295, pH 7.63, and zeta potential +22.6, suggesting liposome stability, and suitability for evaluating leishmanicidal activity. Treatment of infected macrophages with 0.006 nM of 17-AAG:HPβCD or 17-AAG:HPβCD-loaded liposomes resulted in almost complete amastigote clearance inside macrophages after 48 h. This reduction is similar to the one observed in infected macrophages treated with 2 μM amphotericin B. Our results showed that nanotechnology and drug delivery systems could be used to increase the antileishmanial efficacy and potency of 17-AAG *in vitro*, while also resulting in reduced toxicity that indicates these formulations may represent a potential therapeutic strategy against leishmaniasis.

## Introduction

Leishmaniasis, a parasitic disease present on all continents, except Antarctica, is a communicable neglected tropical disease, such as malaria and Chagas disease, which results in 20,000–40,000 deaths per year. Most cases are diagnosed in developing countries, which are associated with poverty, starvation, and poor housing conditions (Alvar et al., 2006, 2012). The *L (L) amazonensis* metacyclic promastigotes embedded in a proteophosphoglycan rich gel located in the most anterior part of the midgut of pool blood feeding phlebotomine flies are regurgitated in the extravascular compartment of the vascularized dermis (Courtenay et al., 2017). Depending on the parasite species and host background, clinical forms can vary from a self-healing lesion at the site of the bite to a disseminated visceral manifestation, with parasites found in the spleen, liver, and bone marrow (Lainson et al., 1987; Yurchenko et al., 2000; Bañuls et al., 2007).

Pentavalent antimonials and amphotericin B (AMB) represent the first line of treatment against leishmaniasis. Antimonials must be administered parenterally, and patients usually undergo more than one treatment in order to achieve parasite clearance (Croft and Olliaro, 2011; Singh and Sundar, 2014; Blum et al., 2018). This protocol results in prolonged treatment periods, which, in conjunction with the occurrence of severe side effects, has led to high rates of treatment abandonment, ultimately favoring the appearance of resistant cases (Croft and Olliaro, 2011; Singh and Sundar, 2014; Blum et al., 2018). AMB, which is administered by intravenous infusion, can cause severe side effects. The liposomal formulation of AMB offers less toxicity and is the most efficient treatment to date, yet its high cost restricts its use in developing countries (Meheus et al., 2010; Croft and Olliaro, 2011; Blum et al., 2018). Second-line drugs, such as pentamidine, and paromomycin, are not used on a large scale due to common side effects and high cost and are only applicable in the treatment of specific cases in which the use of first-line drugs has proven ineffective. The advantage of miltefosine as a second-line drug is its oral route of administration that does not require hospitalization. However, the use of this drug is limited due to teratogenicity and frequent occurrences of therapeutic failure. Taken together, these data highlighted the need to develop new compounds and therapeutic leishmaniasis treatment strategies offering higher efficiency, fewer side effects and decreased cost (Croft and Olliaro, 2011; de Menezes et al., 2015; Zulfiqar et al., 2017; Blum et al., 2018).

Previous studies have demonstrated the potential of heat shock protein 90 (HSP90) as a molecular target for the treatment of parasitic diseases (Banumathy et al., 2003; Kumar et al., 2003; Pallavi et al., 2010; Shonhai et al., 2011; Petersen et al., 2012; Roy et al., 2012; Santos et al., 2014). HSP90 is a molecular chaperone necessary for the correct folding and stabilization of hundreds of proteins, generally called client proteins. Throughout the protozoan life cycle, both in the insect gut and within macrophages in the vertebrate host, HSP90 is activated in response to a variety of stimuli, including stress, changes in temperature and pH, and exposure to an oxidative environment with hydrolytic enzymes (Pallavi et al., 2010; Shonhai et al., 2011).

HSP90 inhibitors, such as 17-N-allylamino-17-dimethoxy geldanamycin (17-AAG), bind to the HSP90 ATP pocket, thereby impairing ATP hydrolysis, and chaperoning activity. Subsequently, unfolded or misfolded client proteins become ubiquitilated and are degraded by the proteasome pathway (Schulte and Neckers, 1998; Isaacs et al., 2003). It has been proven that 17-AAG exerts an antileishmanial effect against both promastigote and intracellular amastigote forms from different *Leishmania* species in a time- and dose-dependent manner, in which parasite cell death ensues without harming macrophage host cells (Petersen et al., 2012; Santos et al., 2014). This occurs independent of macrophage production of microbicidal molecules, such as nitric oxide or superoxide, as well as proinflammatory cytokines, such as TNF-alpha or MCP-1. Also, in a murine model of cutaneous *L. braziliensis* leishmaniasis, 17-AAG was shown to reduce lesion size and parasite load (Santos et al., 2014).

Although 17-AAG is the first HSP90 inhibitor to enter clinical trials as an antineoplastic drug due to significant anti-tumor activity, these have not progressed as a result of hepatotoxicity and nephrotoxicity, as well as poor water solubility and deficient bioavailability (Jhaveri et al., 2014; Wu et al., 2017). These disadvantages led to the need for high dosages to achieve an efficacious therapeutic effect.

Innovative formulations may be able to eliminate the use of toxic excipients, as well as to allow for better drug delivery against *Leishmania* while lessening hepatotoxicity (Shaw and Carter, 2014). One promising strategy is to formulate poorly water-soluble drugs, such as 17-AAG, into nanovectors (Bruni et al., 2017). In the field of nanomedicine, nano-sized drug carriers can offer several pharmacokinetic advantages compared to conventional chemotherapy, such as the controlled release of drugs leading to an improved bioavailability, prolonged activity and significantly less adverse effects (Lamotte et al., 2017). However, a major issue associated with colloidal carriers (i.e., liposomes and nanoparticles) is low water solubility of active compounds, which leads to either decreases in yield during drug loading, or a slow release rate of the drug of interest (Agueros et al., 2011). To overcome these drawbacks, several authors have proposed the use of liposomes containing cyclodextrin (CD) (de Sa et al., 2009). The main purpose of the association between liposomes and CD is to combine the advantages of CD (increased drug solubility) and liposomes (high membrane permeability) into a single dosage form, thereby circumventing the problems associated with each individual system. This cone-shaped compound possesses an internal space that contains glucopyranose units in its molecular structure, as well as hydrogen atoms and glycosidic oxygen bridges that facilitate the entrance of hydrophobic substances at the center of CD cones. External CD surfaces are hydrophilic due to the presence of hydroxyl groups, which increases compound solubility (Kurkov and Loftsson, 2013; Mura, 2014).

Among the many controlled delivery systems, liposomes are the most studied, mainly in the context of artificial membrane models and as drug carriers in *in vivo* models (Gharib et al., 2015). These spherical nanovesicles are composed of lipid bilayers containing one or more aqueous compartments and are thus able to carry hydrophilic and lipophilic molecules. These systems also have the advantage of being biodegradable, biocompatible, non-immunogenic and flexible, depending on composition, size and lipid bilayer fluidity (Anwekar et al., 2011; Wagner and Vorauer-Uhl, 2011). Therefore, liposomes offer an excellent opportunity to encapsulate 17-AAG using the 17-AAG:HPβCD inclusion complex, thusly increasing its pharmacokinetic parameters and potentiating its pharmacological effects. The present study aimed to develop liposomes containing 17-AAG:HPβCD, in addition to characterizing this complex formulation by physicochemical analysis, including X-ray diffraction, and scanning electron microscopy. We also sought to evaluate the stability of different liposomal formulations and, above all, determine the *in vitro* leishmanicidal effect of this liposome-encapsulated inclusion complex.

## Materials and methods

### Chemicals

Solvents, such as chloroform (CHCl_3_) and methanol (CH_3_OH), were purchased from Merck (Darmstadt, Germany). Cholesterol (CHOL), stearylamine (ST), 2-hydroxypropyl-β-cyclodextrin (HPβCD) and 17-(Allylamino)-17-demethoxygeldanamycin (17-AAG, tanespimycin) were purchased from Sigma-Aldrich (St. Louis, USA). Soybean phosphatidylcholine (PC, Lipoid S100®) and lecithin from soy were supplied by Lipoid GmbH (Ludwigshafen, Germanny). All other reagent grade chemicals were obtained from Sigma Aldrich (São Paulo, Brazil).

### Preparation of the inclusion complex

The 17-AAG:HPβCD inclusion complex (17-AAG:HPβCD) was prepared following a Freeze-Drying protocol (Cavalcanti et al., 2011; Cadena et al., 2013).17-AAG and HPβCD were added at a 1:1 molar ratio to a water suspension. The suspension was maintained under agitation at room temperature (25°C) for 72 h, after which a homogenous solution was obtained. The solution was then freeze-dried for 72 h. The 17-AAG:HPβCD inclusion complex was kept protected from light and humidity until use.

### X-ray diffractometry

Powder samples were packed in a x-ray holder, then X-ray diffraction patterns from 17-AAG, HPβCD, and 17-AAG:HPβCD were obtained using a Brucker D8 Advance scanning diffractometer. Data acquisition was performed using radiation (CuKα λ = 1.5406 Å in a 2θ range between 5–60°C with a step size of 0.05° 2θ). Analysis was carried out under ambient conditions. Duplicate determinations were made for each sample.

### Fourier transform infrared spectroscopy (FT-IR)

FT-IR was conducted using a Bruker Vertex v70 FT-IR spectrometer equipped with a diamond attenuated total reflectance accessory (Chandran et al., 2010). The diffuse reflectance technique was in the mid-IR (4000–400 cm^−1^) spectral region, with a resolution of 4 cm^−1^. FT-IR was performed in duplicate for each sample.

### Scanning electron microscopy

Morphology was evaluated by scanning electron microscopy using a Quanta 200F microscope (FEI Company, Hillsboro, OR, USA). Samples (17-AAG, HPβCD, 17-AAG:HPβCD and the physical mixture) were fixed on an aluminum stub using double-sided tape and gold-coated in a vacuum. SEM was performed using an acceleration voltage of 30 kV at a magnification of 600x.

### Liposome preparation

Liposomes containing 17-AAG:HPβCD were prepared using the lipid film method, a procedure well established in literature with high efficiency to obtain small unilamellar vesicles (Akbarzadeh et al., 2013; Cadena et al., 2013). For encapsulation procedures, care was taken to ensure drug stability, including light protection, employment of ultrapure water, sterile beakers and containers, as well as precautions to prevent metal pollution from probe tip during sonication. Briefly, a thin lipid film, denominated as organic phase, consisting of soybean phosphatidylcholine (PC), and cholesterol (CH) with or without stearylamine (SA) in a molar ratio of 7:1.5:0.5 (w/w/w). To obtain liposomal formulation, lipids were added to a mixture of CHCl_3_:Methanol (3:1, v/v) under a magnetic stirring. The solvents were removed under pressure at 37 ± 1°C, 80 rpm using a rotary evaporator (MA120, Marconi). Following the formation of the dry lipid film, an aqueous phase consisting of 10 mL of phosphate buffer solution (pH 7.4) containing 17-AAG:HPβCD was added. The liposomal suspension was then sonicated by a tip ultrasonicator (Q500 from QSonica) at 50% amplitude for 5 min in an ice bath to produce small unilamellar liposomes. 17-AAG:HPβCD was placed in the aqueous phase during lipid film redispersion. The liposomal formulations were then frozen at −80 °C and lyophilized using trehalose as a cryoprotector. Alternatively, fluorescent liposomes were prepared following the protocol described above with the addition of 1 mg of the fluorescent lipid Lumogen F (BASF, Germany) in the organic phase. All preparations were finally suspended in 10 mL of phosphate buffer solution (pH 7.4).

### Characterization of liposomal formulations

#### Liposome stability

Liposomal stability was evaluated using both standard accelerated and long-term stability testing. For the accelerated stability assays, samples were submitted to centrifugation (3,510 × g, for 1 h at 25°C) and mechanical vibration resistance (180 strokes/min for 48 h at 37°C). For long-term stability evaluation, the liposomal formulations were stored at 25°C and evaluated along time intervals (0, 15, and 30 days). After these processes, formulation macroscopic aspects (precipitate formation), pH variation, particle size, and polydispersity index (PDI) were analyzed.

The pH of the liposomes was measured at RT using the glass electrode of a digital pH meter (Orion 5-star plus, Thermo Scientific). Particle size and PDI were determined by standard photon correlation spectroscopy (Beckman Coulter Delsa™ Nano S Particle Analyzer). All liposomal formulations were previously diluted in ultrapure water (1:5, liposomes: water) and measured at 25°C at a fixed 90° angle.

#### Encapsulation efficiency of 17-AAG into liposomes

The encapsulation efficiency (EE%) of 17-AAG into liposomes was determined by the ultrafiltration-centrifugation technique using Ultrafree^®;^ units (Millipore, USA). After centrifugation of the liposomal samples at 8,776 × g for 1 h at 4°C, 17-AAG content was measured by UV spectrophotometry using an analytical method to determine the concentration of 17-AAG that shows the highest peak of absorbance. For this, methanol solutions of 17-AAG at concentrations that vary from 1 to 9 μg/mL were read at a variety of wavelengths ranging from 200 to 500 nm. We found that the highest absorbance of the 17-AAG solution was detected at λmax 333 nm (Supplementary Figure 1). Experiments were performed in triplicate. EE% was then calculated as follows:

$$\begin{array}{l} {\%EE}_{17-AAG}=\left(\left[measured_{17-AAG}\right]-\left[unloaded_{17-AAG}\right]\right)\\ ÷\left[tmeasured_{17-AAG}\right]\;\times 100 \end{array}$$

#### Zeta potential

The zeta potential (ζ, mV) of liposomes was measured using a dynamic light scattering technique (Zetasizer Nano ZS, Malvern, UK) to analyze surface charge. All liposomal formulations were previously diluted in ultrapure water (1:5 liposomes:water) and measurements were performed in triplicate.

#### Transmission electron microscopy/TEM liposome morphological analysis

The evaluation of liposome morphology was performed by transmission electron microscopy (TEM). Liposomal formulations were first diluted in pure water (1:10) and dripped (10 μL) onto a grid containing a quadratic copper network coated with a carbon film. After 5 min, the excess sample was removed by rapid transfer on a filter followed by negative staining with 10 μL of phosphotungstic acid solution (1%, v/v) for 5 min. After 24 h, microphotographs were captured using a transmission electron microscope (Tecnai, 200, FEI).

### *Leishmania* parasites

*Leishmania (L) amazonensis* (MHOM/BR/87/BA125) parasites were maintained by serial passage in C57BL/6 mice to preserve parasite infectivity. Parasites were isolated from the popliteal lymph nodes of infected mice and axenic parasites were maintained by serial passages in Schneider's insect medium supplemented with 10% FCS and 50 μg/mL of gentamycin. Axenic cultures were maintained for a maximum of seven passages.

### Cytotoxicity against macrophages

Inflammatory macrophages were obtained through peritoneal lavage 4 days after the intraperitoneal injection of thioglycolate in CBA mice as previously described by Gomes and coworkers (Gomes et al., 2003). Macrophages were maintained in DMEM complete medium (DMEM medium supplemented with 10% inactivated FCS, 2 g/L of sodium bicarbonate, 25 mM HEPES, 1 mM of glutamine and 0.2% of ciprofloxacin) at 37°C under 5% CO_2_ and 95% humidity. Cells were plated at a concentration of 5 × 10^4^ cell per well on 96-well plates for 12–18 h. The cytotoxicity of different 17-AAG formulations was measured using an AlamarBlue^®;^ assay (Thermo Fisher Scientific, USA) in accordance with manufacturer instructions. Briefly, macrophages were incubated for 72 h with 12-step serial dilution concentrations (start value of 1 nM) of unloaded liposomes, 17-AAG:HPβCD-loaded liposomes or 17-AAG:HPβCD alone. Next, AlamarBlue was added to the wells at a final concentration of 10% and cells were reincubated for another 18–24 h. Reagent absorbance was then measured at wavelengths of 570 and 600 nm using a spectrophotometer (SPECTRA Max 340 PC). Untreated cells were used as controls considering 100% viability.

### Anti–promastigote activity

Axenic promastigotes of *L. (L) amazonensis* were plated at a final concentration of 4 × 10^5^ cells per well on a 96-well plate and incubated for 72 h with 12-serial dilution concentrations (start value of 1 nM) of unloaded liposomes, 17-AAG:HPβCD-loaded liposomes, or 17-AAG:HPβCD alone. Next, AlamarBlue was added to the wells at a final concentration of 10% and cells were reincubated for another 4–6 h. Reagent absorbance was then measured at wavelengths of 570 and 600 nm using a spectrophotometer (SPECTRA Max 340 PC). Untreated cells were used as controls considering 100% viability.

### Anti-intracellular parasite activity

To assess the treatment effect on infection, we plated macrophages on glass coverslips and then infected with promastigotes in the stationary phase of growth at 35°C under 5% CO_2_ and 95% humidity for 6 h, following washout to remove any non-internalized parasites. Infected macrophages were then incubated for additional periods of time accordingly to the following protocols: (i) To determine the percentage of infected macrophages at early stage of infection, macrophages were then incubated for an additional 48 h, followed by treatment with 0.006 nM of unloaded liposomes, 17-AAG:HPβCD, or 17-AAG:HPβCD-loaded liposomes for 48 h, and finally fixed with formaldehyde 4% for 20 min at room temperature. The slides were then mounted using ProLong Gold (Molecular Probes, OR, USA) with DAPI for nuclei staining. The percentage of infected macrophages was determined by counting DAPI stained parasite nuclei in the interior of at least 400 cells per slide under a fluorescent microscope (Olympus BX 51). Experiments were performed at least twice in sextuplicate. (ii) To assess intracellular parasite viability, infected macrophages were treated at different times after infection—immediately after 6 h of infection and washout of non-internalized parasites (very early time) or, after an additional incubation time of 72 h (late time after infection), the time necessary for internalized promastigotes to differentiate into amastigote forms. After both time points, macrophages were treated with 0.006 nM of unloaded liposomes, 17-AAG:HPβCD-loaded liposomes, 17-AAG:HPβCD, 17-AAG for additional 48 h. AMB was used as a parasite death control at the concentration of 0.006 nM in cultures incubated for 6 h and at 2 μM in those cultures treated for 72 h. At the end of incubation times, all cultures were washed thrice and then washed medium was replaced with 1 mL of fresh Schneider's complete medium. Parasites that remain viable were liberated in the medium and incubated at 23°C for additional 5 days. Parasite viability was assessed by quantification of the number of amastigotes that had transformed into motile promastigotes was performed. All experiments were performed at least twice in sextuplicate.

### Uptake of fluorescent liposomes by infected macrophages

Inflammatory peritoneal macrophages were obtained as described above and plated at a concentration of 10^5^ cells per well on a 24-well plate with glass coverslips. Prior to macrophage infection, *L. (L) amazonensis* parasites were stained with carboxyfluorescein (CSFE) to obtain green fluorescent parasites. Briefly, 10^8^ parasites were incubated in 4 mL of saline solution with 1 μM of CFSE for 10 min at 37°C. Next, 4 mL of FBS was added and reincubated for an additional 1 min. The parasites were then washed thrice and the plated macrophages (10:1) were infected for 4 h. All plates were subsequently washed to remove any non-internalized parasites and then reincubated for an additional 72 h. Next, cells were treated with fluorescent liposomes for 30 min, washed and then a group of cells was fixed with 4% paraformaldehyde for 20 min at room temperature. Three other groups were then reincubated for an additional 30, 120, or 240 min, followed by fixing. Coverslips were then mounted on slides using ProLong Gold with DAPI for nuclear staining. Images were captured on a Leica SP8 confocal microscope. Fluorescence and differential interference contrast (Panzitta et al., 2015) images were acquired using resonant scanning mode at a resolution of 1,024 × 1,024 under magnification (63 ×) with an oil immersion objective and z-stacks ranging from 0.3 to 0.5 μm.

### Statistical analysis

Graphing and statistical analysis were performed using the GraphPad Prism program (version Mac v6.0). Kolmogorov-Smirnov test was used for assessing the normality of the data. For Gaussian distributed data, Student's *t*-test and one-way ANOVA followed Dunn's Multiple Comparison tests were used for comparisons between two groups or among three or more groups, respectively, while the Mann-Whitney and the Kruskal-Wallis non-parametric tests were used for comparisons between two groups and among three or more groups, respectively. CC50 and IC50 values were calculated using GraphPad Prism software v6.0. Data were normalized and then subjected to nonlinear regression analysis (curve fitting; de Sa et al., 2009). Differences among groups were considered statistically significant when *p* < 0.05.

### Ethics statement

Male and female CBA and C57BL/6 mice were provided by the IGM/FIOCRUZ Animal Care Facility. All animals were housed under specific pathogen-free conditions, fed commercially available rations and given water *ad libitum*. Mice were euthanized at 8–12 weeks of age. All protocols for animal handling and experimentation were performed in accordance with the International Guiding Principles for Biomedical Research Involving Animals and this research proposal received approval from the IGM Institutional Review Board for Animal Experimentation.

## Results

Preparation and characterization of the 17-AAG:HPβCD inclusion complex

**Figure 1:**
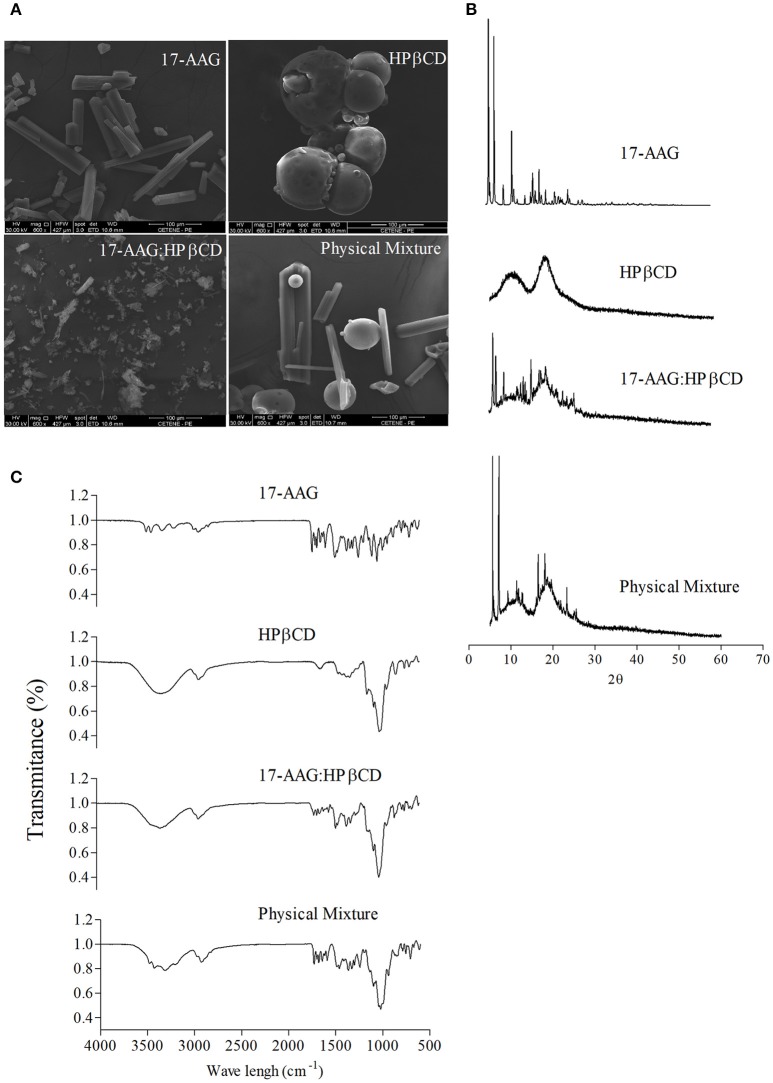
Characterization of the 17-AAG:HPβCD inclusion complex. **(A)** SEM of 17-AAG; HPβCD; 17-AAG:HPβCD and the physical mixture. Magnification: 600 X. **(B)** X-ray diffraction patterns of 17-AAG; HPβCD; 17-AAG:HPβCD and the physical mixture. **(C)** FT-IR spectra of 17-AAG; HPβCD; 17-AAG:HPβCD and physical mixture.

Using a calibration curve constructed with different concentrations of 17-AAG at λ_max_ = 333 nm (Supplementary Figure 2) revealed that the synthesis of the 17-AAG:HPβCD complex by lyophilization obtained an efficiency of over 90% with an overall increase of 33 times in water solubility (Supplementary Figures 3, 4). Next, we evaluated the morphology of 17-AAG, HPβCD, 17-AAG:HPβCD, and the physical mixture by SEM (Figure 1A). Photomicrographs revealed that 17-AAG possesses a crystal-like structure, while HPβCD presents a bead-like appearance. The physical mixture consisted of two distinct forms, equal to pure compounds, of differing size and shape on a uniform scale. With regard to 17-AAG:HPβCD, a single phase with an irregular shape was identified, unlike pure 17-AAG or HPβCD.

X-ray diffraction patterns for 17-AAG, HPβCD, 17-AAG:HPβCD, and the physical mixture are depicted in Figure 1B. We observed that 17-AAG presented crystalline structure characteristics with several peaks of high intensity, whereas an amorphous pattern lacking crystalline peaks was observed for HPβCD. The diffractogram of the physical mixture revealed peaks similar to those corresponding to the pure components, 17-AAG and HPβCD, indicating that no interaction occurred between these two components. Concerning 17-AAG:HPβCD, diffraction analysis revealed sharp peaks similar to those observed for 17-AAG, albeit of lower intensity, indicating interaction between the 17-AAG and HPβCD molecules.

The FT-IR spectra of 17-AAG, HPβCD, the 17-AAG:HPβCD, and the physical mixture are shown in Figure 1. Relatively sharp bands were recorded for 17-AAG in the region of 3,700–3,100 cm^−1^. These bands exhibited a shortening when 17-AAG was complexed to HPβCD. Displacements and absence of typical bands of 17-AAG were also observed at 1,730–1,042 cm^−1^. For example, it can be observed that the C = O band at 1,730 cm^−1^ was displaced to 1,712 cm^−1^ in the spectrum of 17-AAG:HPβCD. Still, the absorption peak of 1,519 cm^−1^ corresponding to the C = C group represents the elongation vibration of the aromatic ring, which exhibited decreased intensity in the inclusion complex spectrum. The 17-AAG spectrum also revealed that peaks corresponding to the CH_3_ group at 1,486, 1,463, and 1,413 cm^−1^, as well as bands at 1,099 and 1,045 cm^−1^, corresponding to C = H of the benzene ring, were not evident in 17-AAG:HPβCD spectrum. We also used thermal analysis (differential scanning calorimetry and thermogravimetry) to characterize 17-AAG:HPβCD, which revealed the inclusion complex was formed (Supplementary Figure 5).

### Characterization of 17-AAG:HPβCD-loaded liposomes

#### Stability of liposomal formulations

Samples of unloaded and 17-AAG:HPβCD-loaded liposomes were subjected to accelerated and long-term stability testing. With regard to accelerated stability (Table 1), both of the liposomal formulations maintained their initial characteristics (particle size, PDI, and pH) in response to mechanical stress and centrifugation. With regard to long-term stability (Table 2), the suspended liposomal forms were observed to remain stable for 30 days. In addition, no differences in stability were observed in the unloaded and 17-AAG:HPβCD-loaded liposomes in any of the evaluated parameters. Importantly, all evaluated liposomes exhibited macroscopic characteristics of stable liposomal suspensions, without any apparent lumps or precipitations.

**Table 1:** Accelerated stability testing of unloaded and 17-AAG:HPβCD-loaded liposomes.

**Formulation**		**Parameters**		
		**Ø (nm)**	PDI^*^	**pH**
Mechanical Stirring (180 strokes/min, 48h, 37 °C)	Unloaded liposome	115.3 ± 2.03	0.246	7.50
	17-AAG:HPβCD- loaded liposome	127.2 ± 0.16	0.295	7.63
Centrifugation (3,510 × g, 1 h, 4°C)	Unloaded liposome	131.0 ± 2.65	0.313	7.46
	17-AAG:HPβCD- loaded liposome	145.3 ± 6.73	0.508	7.63

*Ø, particle size*;

* *PDI, Polydispersity index*.

*Data are presented as mean ± standard deviation*.

**Table 2:** Long-term stability testing of unloaded and 17-AAG:HPβCD-loaded liposomes.

**Formulation**	**Parameters**								
	**0 days**			**15 days**			**30 days**		
	**Ø (nm)**	PDI^*^	**pH**	**Ø (nm)**	PDI^*^	**pH**	**Ø (nm)**	PDI^*^	**pH**
Unloaded liposome	130.7 ± 1.82	0.308	7.61	131.6 ± 2.35	0.309	7.64	141.9 ± 1.10	0.324	7.62
17-AAG:HPβCD-loaded liposome	157.4 ± 3.75	0.352	7.63	163 ± 1.45	0.323	7.5	169 ± 2.14	0.315	7.5

*Ø, particle size*;

* *PDI, Polydispersity index*.

*Data are presented as mean ± standard deviation*.

#### Morphological analysis

**Figure 2:**
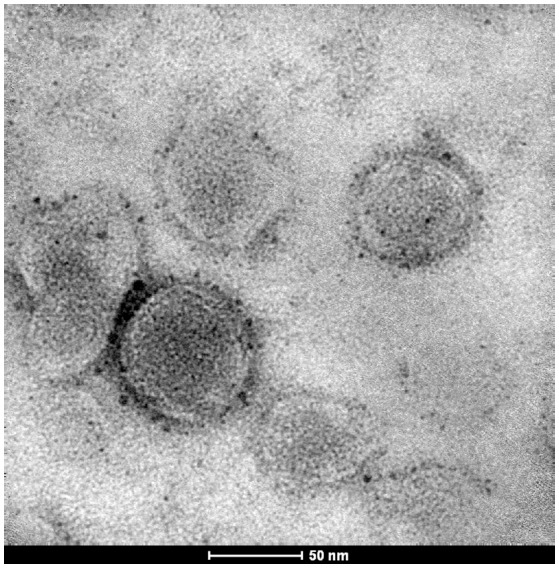
Transmission electron microscopy of 17-AAG:HPβCD-loaded liposomes.

Transmission electron microscopy (TEM) revealed that liposomes containing 17-AAG:HPβCD displayed several concentric vesicles, of sizes smaller than 220 nm (Figure 2).

#### Content, zeta potential, and encapsulation efficiency

Table 3 lists the characteristics of size, zeta, potential and encapsulation efficiency of the liposomes containing 17-AAG:HPβCD. Zeta potential values were similar between the unloaded and 17-AAG:HPβCD-loaded liposomes, ranging from + 21.1 ± 0.7 to + 22.6 ± 1.9 mV, respectively. In addition, liposomes containing 17-AAG:HPβCD presented high 17-AAG loading (89.2 ± 4.4%) and encapsulation efficiencies (99.5 ± 0.3%).

**Table 3:** Characterization of liposomes: content (%), percentage of encapsulation efficiency (EE%) and zeta potential.

**Formulation**	**Parameters**		
	**Content (%)**	**EE (%)**	**Zeta potential (mV)**
Unloaded liposome	–	–	+ 21.13 ± 0.68
17-AAG:HPβCD-loaded liposome	89.23 ± 4.36	99.53 ± 0.30	+ 22.6 ± 1.85

*Data are presented as mean ± standard deviation*.

### Effects of liposomal formulations on promastigotes and host cells

**Figure 3:**
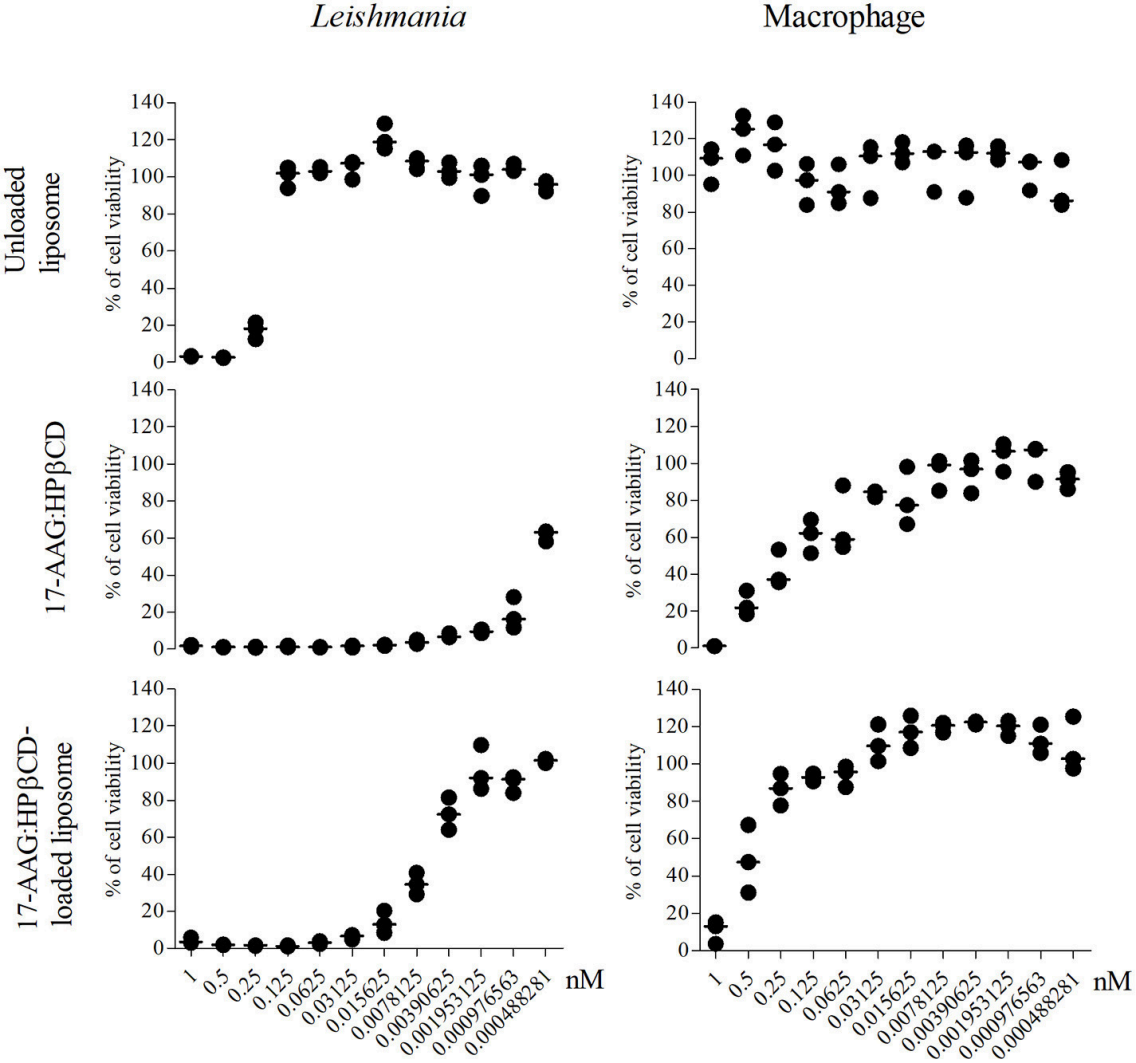
Nanoformulations of 17-AAG reduced the viability of axenic *Leishmania* promastigotes. Macrophages and log-phase promastigotes of *L. (L) amazonensis* were treated with 12-step serial dilutions (1:2) of unloaded liposomes; 17-AAG:HPβCD inclusion complex and 17-AAG:HPβCD-loaded liposomes for 72 h. Each graph is representative of one out of three experiments performed in triplicate. Lines are representative of median values from a single experiment.

After the preparation and characterization of the liposomal formulations containing the inclusion complex, we sought to investigate potential effects against *Leishmania* promastigotes and evaluate toxicity to macrophages, i.e., mammalian host cells. At the concentrations evaluated, unloaded liposomes presented toxicity to *Leishmania* only at higher concentrations, e.g., 1.0, 0.5, and 0.25 nM (Figure 3). On the other hand, 17-AAG:HPβCD and 17-AAG:HPβCD-loaded liposomes demonstrated toxicity to *Leishmania* at very low concentrations: 0.0078 and 0.00097 nM, respectively (Figure 3). Moreover, while macrophages treated with unloaded liposomes presented no reduction in viability, treatment with 17-AAG:HPβCD or 17-AAG:HPβCD-loaded liposomes was shown to reduce cell viability only at the highest tested concentrations: 0.5 and 0.125, respectively (Figure 3).

It is noteworthy that the IC_50_ calculated for 17-AAG:HPβCD-loaded liposomes was 0.0061 (Q1: 0.0041; Q3: 0.0076) nM, while the CC_50_ calculated for macrophages was 0.48 (Q1: 0.24; Q3: 0.77) nM. This resulted in a selectivity index value (SI = 0.48 nM/0.0061 nM) that indicates the 17-AAG:HPβCD-loaded liposomes present 79.36 times greater toxicity against *Leishmania* in comparison to host cells.

### Liposome effects on intracellular parasite

**Figure 4:**
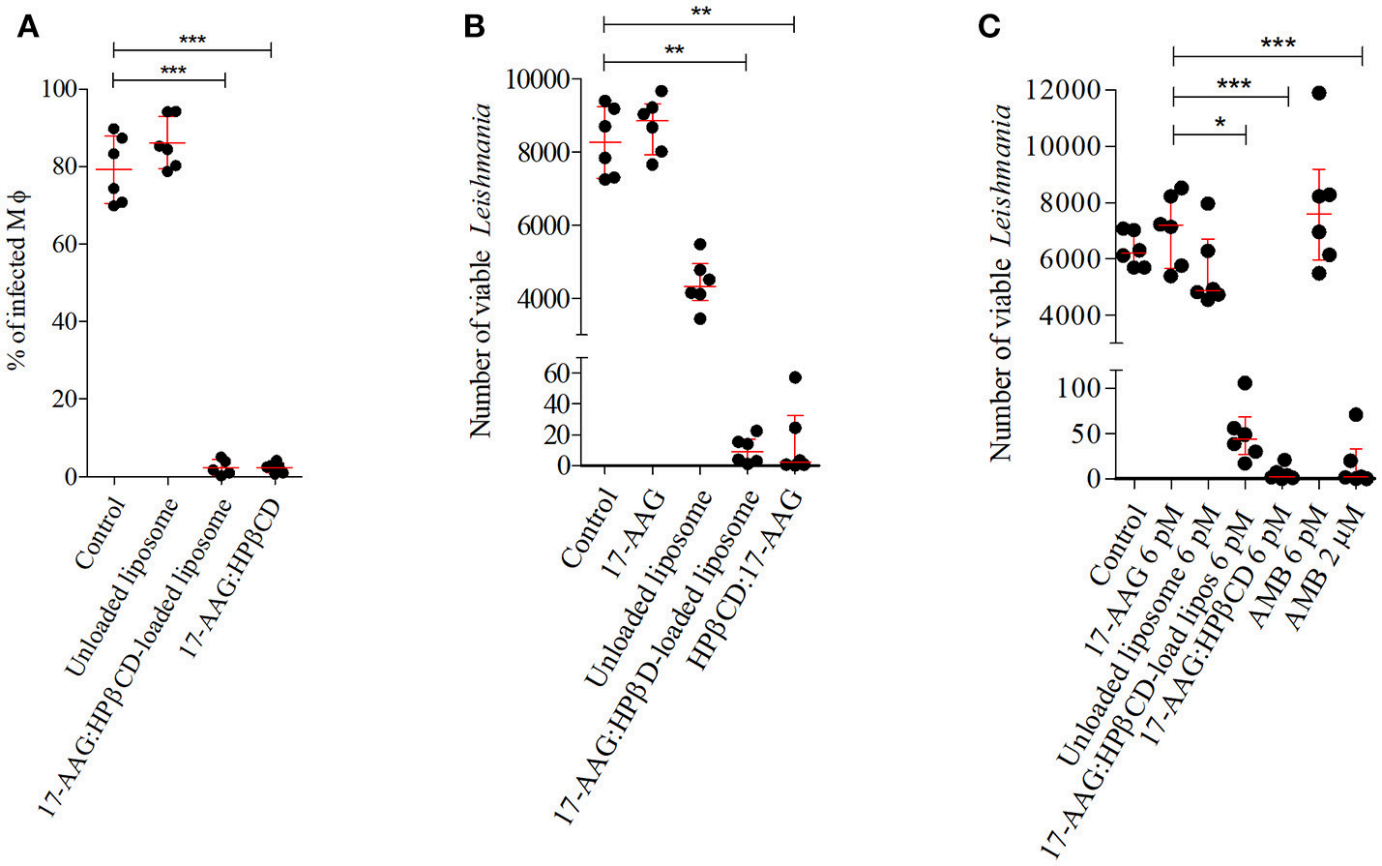
Effect of 17-AAG nanoformulations on intracellular *L. (L) amazonensis*. **(A)** Percentage of infected macrophages at earlystage of infection. Infected macrophages were treated with 0.006 nM of unloaded liposomes; 17-AAG:HPβCD inclusion complex and 17-AAG:HPβCD-loaded liposomes for 48 h. Cells were fixed, DAPI stained and analyzed under fluorescent microscopy. At least 400 cells were counted per coverslip. **(B)** Intracellular parasite viability at very early stages of infection. Macrophages were infected for 6 h and then treated with 0.006 nM of 17-AAG:HPβCD-loaded liposomes compared to controls: pure 17-AAG; unloaded liposomes; 17-AAG:HPβCD for 48 h. **(C)** Intracellular parasite viability at later stages of infection. Macrophages were infected for 72 h and then treated with 0.006 nM of the following compounds: 17-AAG, unloaded liposomes, 17-AAG:HPβCD, 17-AAG:HPβCD-loaded liposomes and AMB, and 2 μM AMB for 48 h. **(A)** Dots represent individual replicates for a group of treated macrophages. Lines represent means ± SD of one representative experiment out of three performed in sextuplicate (one-way ANOVA, Dunn's Multiple Comparison Test, ***p < 0.0001). **(B,C)** Dots represent individual replicates for a group of treated macrophages. Lines represent median ± interquartile range (25% and 75%) for one representative experiment out of two performed in sextuplicate (Kruskal-Wallis test, Dunn's Multiple Comparison test, *p < 0.05, **p < 0.01, ***p < 0.001).

After confirming increased toxicity of 17-AAG:HPβCD inclusion complex-loaded liposomes against *Leishmania* compared to macrophages, we attempted to investigate the potential effects of these liposomes against intracellular parasites. Infected macrophages treated with 0.006 nM (Calculated IC_50_) of 17-AAG:HPβCD-loaded liposomes and 0.006 nM of 17-AAG:HPβCD for 48 h. The percentage of infected macrophages treated with unloaded liposomes (86.09 ± 6.65 %) was not different from that of control untreated cells (79.2 ± 8.67 %; *p* > 0.005; Figure 4A). Treatment with 17-AAG:HPβCD-loaded liposomes and 17-AAG:HPβCD reduced the percentage of infected cells to 2.4 ± 2.02 and 2.3 ± 1.28 %, respectively, which corresponded to reductions of 96.9 and 97.09% when compared to untreated control cells (*p* < 0.0001, Figure 4A).

We then conducted experiments to investigate intracellular parasite viability after treatment with 17-AAG:HPβCD-loaded liposomes in comparison to controls, unloaded liposomes and 17-AAG:HPβCD. Treatment of infected macrophages with 0.006 nM of 17-AAG or unloaded liposomes resulted in no differences in the number of viable parasites, with median values of 8,850 (Q1: 7,920 to Q3: 9,323), and 4,330 (Q1: 3,943 to Q3 4,948) parasites/mL, respectively, when compared to untreated control cells: 8,265 (Q1: 7,279 to Q3: 9,233) parasites/mL (*p* > 0.005, Figure 4B). On the other hand, treatment of infected cells with 0.006 nM of 17-AAG:HPβCD-loaded liposomes and 17-AAG:HPβCD drastically reduced the number of viable parasites to median values of 9 (Q1: 2.5 to Q3: 17.25) and 2 (Q1: 0.31 to Q3: 32.63) parasites/mL, respectively, compared to control cells. In addition, when macrophages were treated at 72 h after infection, a sufficient time to allow for the complete differentiation of *Leishmania* into its intracellular amastigote form, treatment with 17-AAG, unloaded liposomes or AMB, all compounds at a concentration of 0.006 nM, did not alter the intracellular parasite viability in comparison to the control untreated group. The resulting median numbers of viable parasites were 7,185 parasites/mL (Q1: 5,670 to Q3: 8,295) for 17-AAG, 4,880 parasites/mL (Q1: 4,595 to Q3: 6,700) for unloaded liposomes and 7,590 parasites/mL (Q1: 5,985 to Q3: 9,180) for AMB, which were similar to what was seen in untreated control cells: 6,210 (Q1: 5,700 to Q3: 7,035) parasites/mL (*p* > 0.005, Figure 4C). On the other hand, infected cells treated with 17-AAG:HPβCD-loaded liposomes and 17-AAG:HPβCD significantly reduced the number of viable parasites to 43.75 (Q1: 23.75 to Q3: 68.50) and 2.75 (Q1: 0.75 to Q3: 10.88) parasites/mL, respectively, vs. 7,185 ranging from Q1: 5670 to Q3: 8295 parasites/mL observed in cells treated with 0.006 nM of 17-AAG (*p* < 0.005, Figure 4C). This reduction is comparable to what was observed in the positive control group treated with 2 μM of AMB: 2 (Q1: 0 to Q3: 32.75) parasites/mL.

**Figure 5:**
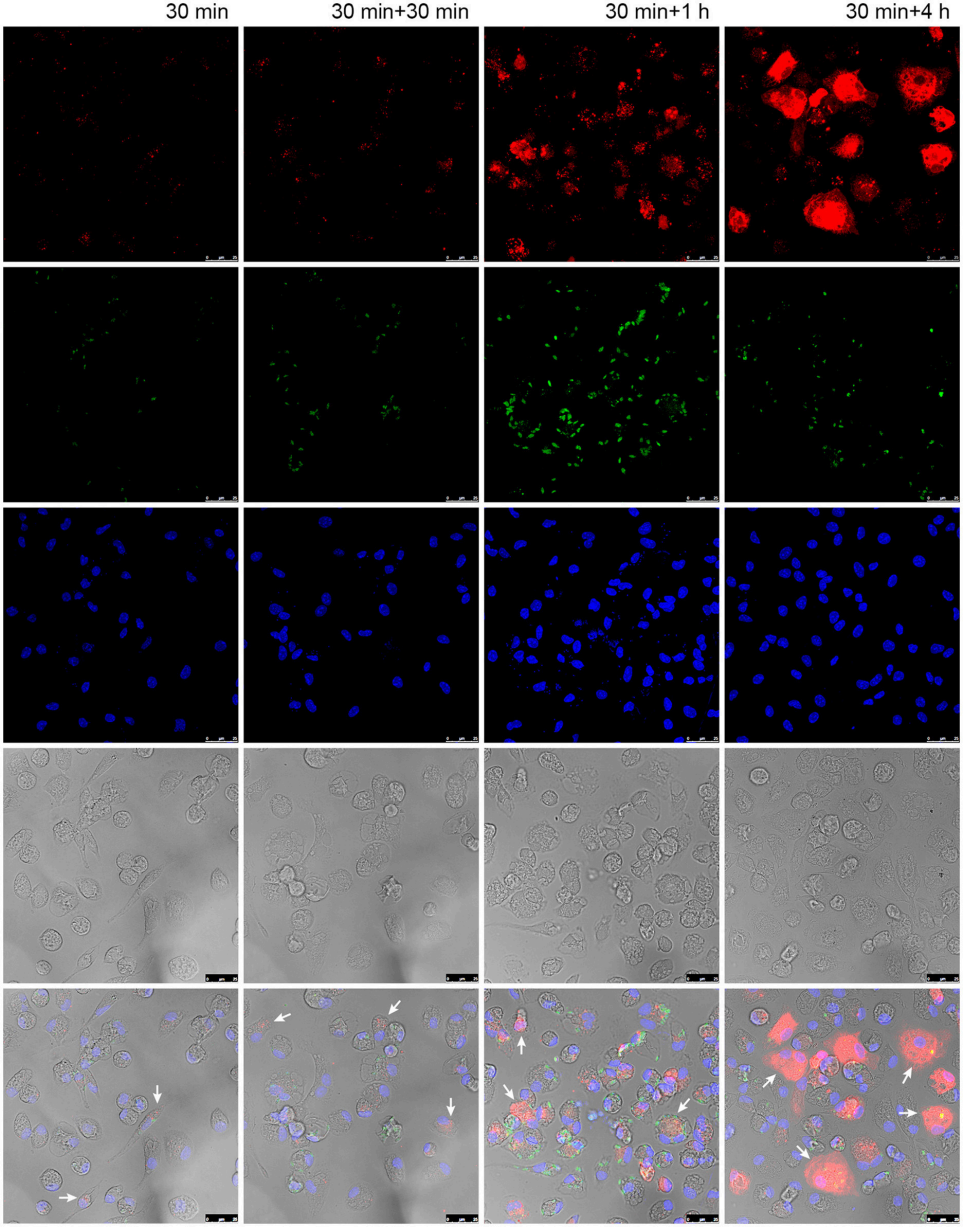
Liposome uptake in macrophages infected with *L. (L) amazonensis*. Peritoneal macrophages from CBA mice were infected with *L. (L) amazonensis* promastigotes for 4 h. Cells were then washed to remove non-internalized parasites and reincubated for an additional 72 h. After that time, cells were treated with fluorescent Lumogen F-liposomes for 30 min and then fixed or reincubated for additional 30, 120, and 240 min. Images were acquired using a confocal microscope. Arrows: infected macrophages containing fluorescent liposomes. Red, Lumogen F-liposomes, green, CSFE stained *L. (L) amazonensis*; blue, DAPI DNA stain; gray, DIC. The images shown are representative of two independent experiments.

We were also interested in evaluating the kinetics of liposome uptake in infected macrophages. Time-dependent trafficking of liposome was evaluated by confocal microscopy (Figure 5). The liposomes were rapidly internalized after 30 min of incubation and continued to accumulate in macrophage cytoplasm up to 4 h after liposome treatment. It is noteworthy that in shorter time points liposome appear as heterogeneous spots, while at later time points it is observed a diffuse red pattern covering large portions of the macrophage cytoplasm.

## Discussion

We previously demonstrated that 17-AAG, an HSP90 inhibitor, is capable of killing axenic promastigotes and intracellular amastigotes of *Leishmania*. This compound was also shown to reduce lesion size and parasite burden at the site of infection in a murine model of cutaneous leishmaniasis (Petersen et al., 2012; Santos et al., 2014). Despite the substantial *in vitro* and *in vivo* activity exhibited by 17-AAG, its poor water solubility and bioavailability represent major drawbacks in applicability (Egorin et al., 2002).

More and more studies are conducted to optimize therapeutic index of small component such as 17-AAG /tanespimycin, an inhibitor of the otherwise ubiquitous HSP-90 a protein recognized as a highly abundant and essential molecular chaperone protein in both unicellular and multicellular eukaryotes (Calderwood and Prince, 2018). To the best of our knowledge, the present study represents the first attempt to utilize nanotechnology in the delivery of 17-AAG for leishmaniasis treatment.

For preparing liposome-containing 17-AAG, first we incorporated 17-AAG to HPβCD inclusion complex which yielded a 33-fold increase in 17-AAG water solubility (Supplementary Figures 3, 4). 17-AAG content into inclusion complexes was estimated by dissolving a fixed amount of the complexes in methanol. Appropriate dilutions were made, and the 17-AAG content was calculated from UV absorbance recorded at λ max 333 nm, wavelength previously used to detect 17-AAG in polymer micelles (Jelonek et al., 2016). As described in Material and Methods section, 17-AAG concentration into liposomes was also determined using the ultrafiltration-centrifugation technique using Ultrafree^®;^ units. These centrifugal devices with disposable filters ate known to enable separation of free 17-AAG (unloaded drug) from liposomal suspension.

The characterization of the 17-AAG:HPβCD inclusion complex by SEM revealed an amorphous irregular shape, distinct from both isolated compounds, which is consistent with previous descriptions of other compounds submitted to complexation to HPβCD (Cavalcanti et al., 2011). To further characterize the 17-AAG:HPβCD inclusion complex, X-ray diffraction analysis exhibited a crystalline pattern similar to what was observed in pure 17-AAG, albeit with lower intensity as a result of chemical interactions between the two complexed components. The behavior of the inclusion complex suggests the formation of a new solid phase as opposed to simply physically mixing of its individual components, which is consistent with reports described for other compounds (Liu et al., 2006; Saxena et al., 2012). Furthermore, characterization of 17-AAG:HPβCD by infrared analysis (Szente, 1996) revealed that 17-AAG indeed established molecular interactions with HPβCD. The displacement or absence of typical bands in the spectra of pure 17-AAG compared to those of the 17-AAG:HPβCD could be interpreted as a shift in the hydrogen bonds between 17-AAG and cyclodextrin (Misiuk and Jozefowicz, 2015). Of note, hydroxyl groups for HPβCD might interact with the carbonyl group of 17-AAG, and shortening of 17-AAG bands confirms the formation of such interactions by hydrogen bondings (Misiuk and Jozefowicz, 2015). Therefore, these findings collectively indicate that changes on characteristic peaks of 17-AAG might be attributed to the inclusion of this drug within the HPβCD cavity. Also differential scanning calorimetry and thermogravimetry analysis were performed and corroborate the formation of the inclusion complex (Supplementary Figure 5). Even though the complexation of 17-AAG with HPβCD increased the solubility of 17-AAG and, this complex does not constitute a delivery system against intracellular parasites (Challa et al., 2005; Loftsson et al., 2007). For this reason, we incorporated 17-AAG:HPβCD inside liposome vesicles, which provide controlled and sustained drug release (Anwekar et al., 2011). First, unloaded and 17-AAG:HPβCD-loaded liposomes were subjected to accelerated and long-term stability testing (Cadena et al., 2013). No significant alterations in the parameters evaluated, such as particle size, PDI and pH, were found in unloaded or 17-AAG:HPβCD-loaded liposomes, which attests to the stability of the evaluated nanoformulations. In addition, particle size also remained unaltered after stability testing, as TEM revealed a liposome size that presents ideal pharmacokinetic behavior, since liposomes smaller than those incorporated into 17-AAG:HPβCD can result in rapid renal clearance, while larger sizes can lead to swift hepatic clearance (Panzitta et al., 2015).

The complexation of insoluble compounds with CD, and the incorporation of these into liposomes, has been proven to enable reductions in the therapeutic concentrations of insoluble compounds (Loftsson et al., 2007; Bruni et al., 2017). It was demonstrated in a murine model of leishmaniasis that meglumine antimoniate-β-CD presents increased oral bioavailability, potency, and efficiency in comparison to meglumine antimoniate alone (Demicheli et al., 2004; Frézard et al., 2008). Moreover, the β-CD-17AAG complex exhibited enhanced toxicity against T47D epithelial-like breast cancer cells in comparison to free 17-AAG (Ghalhar et al., 2014). Similarly, Roychoudhury and co-workers (Roychoudhury et al., 2011) observed a reduction of up to 97% in parasite burden compared to controls using sodium stibogluconate entrapped in phosphatidylcholine stearylamine-bearing liposomes as opposed to sodium stibogluconate alone (Roychoudhury et al., 2011). Our results regarding the uptake of liposome by *Leishmania*-infected macrophages corroborates the previews findings of Borborema and co-workers (Borborema et al., 2011) that liposomes were quickly internalized after 30 min of interaction and continued to localize in the cytoplasm for at up to 4 h. The persistence availability of liposomal content in macrophage host cells even after cell wash may be an important factor that contributes to parasite clearance.

The increased leishmanicidal efficacy of liposomes encapsulated with 17-AAG:HPβCD was evidenced by the comparatively low median IC_50_ value of these liposomes in comparison to the IC_50_ of pure 17-AAG. The treatment of infected macrophages with the corresponding IC_50_ concentration of liposomes containing 17-AAG:HPβCD remarkably reduced the percentage of infected cells and the number of parasites per cell in comparison to untreated or unloaded liposome-treated macrophages. This nanoformulation also significantly reduced parasite intracellular viability at concentrations much lower than what is employed using the leishmanicidal agent AMB *in vitro*, which is known to exert a similar antileishmanial effect. Similar reductions in intracellular parasite viability were only observed when pure 17-AAG was employed at a concentration 80% higher than the corresponding IC_50_ concentration of the liposomal formulation (Petersen et al., 2012; Santos et al., 2014).

Our results provide evidence of the intermolecular interaction between 17-AAG and HPβCD via the formation of the 17-AAG:HPβCD inclusion complex. Our data also show that the encapsulation of 17-AAG:HPβCD inside liposomes results in high antileishmanial efficiency. More importantly, the data presented herein corroborate our group's hypothesis that treatment with 17-AAG represents a promising therapeutic strategy for the elimination of intracellular *Leishmania* (Petersen et al., 2012; Roy et al., 2012; Santos et al., 2014). Considering the fact that other HSP90 inhibitors have entered clinical trials, it is our hope that further *in vivo* experimentation will elucidate the leishmanicidal potential of this 17-AAG nanoformulation.

## Ethics statement

This study was carried out in accordance with the recommendations of Guiding Principles for Biomedical Research Involving Animals. The protocol was approved by the Instituto Gonçalo Moniz Institutional Review Board for Animal Experimentation.

## Author contributions

AP, TC, JVdM, GM, and PV conceived and designed the experiments. AP, TC, DD, JdS, and JdM performed the experiments. AP, TC, JdM, FF, and PV analyzed the data. JdM, GM, FF, and PV contributed to reagents, materials, analysis tools. AP, TC, JR, and PV wrote the paper.

### Conflict of interest statement

The authors declare that the research was conducted in the absence of any commercial or financial relationships that could be construed as a potential conflict of interest. The reviewer GM and handling editor declared their shared affiliation at time of review.
