# Supplementary figures and images for: Encapsulation of the HSP-90 Chaperone Inhibitor 17-AAG in Stable Liposome Allow Increasing the Therapeutic Index as Assessed, in vitro, on Leishmania (L) amazonensis Amastigotes-Hosted in Mouse CBA Macrophages
Source: Front Cell Infect Microbiol. 2018 Aug 30;8:303. doi: 10.3389/fcimb.2018.00303 (PMC6126448; doi:10.3389/fcimb.2018.00303)

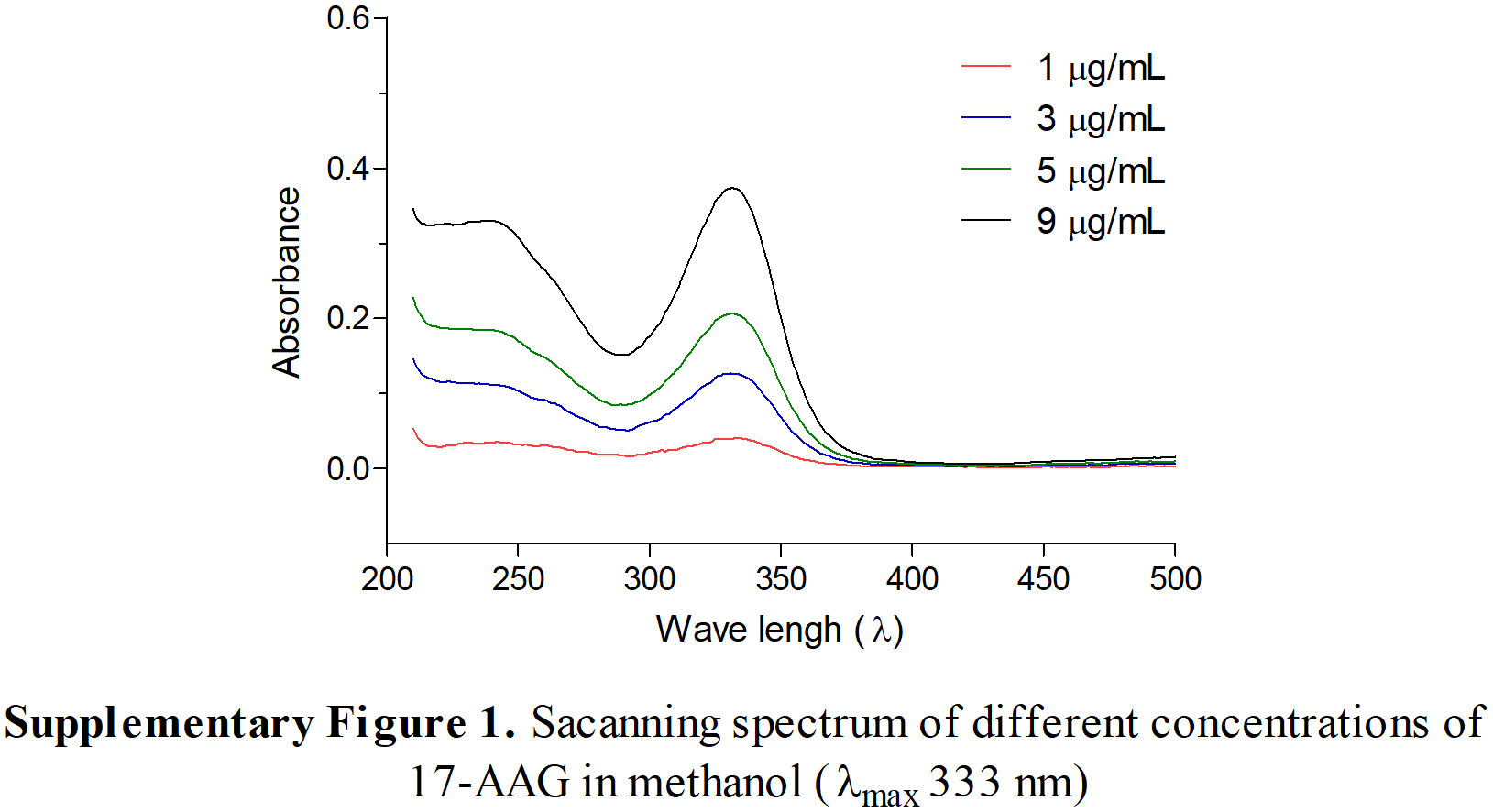

Supplement: Supplementary file 1 [file Image_1.TIF]

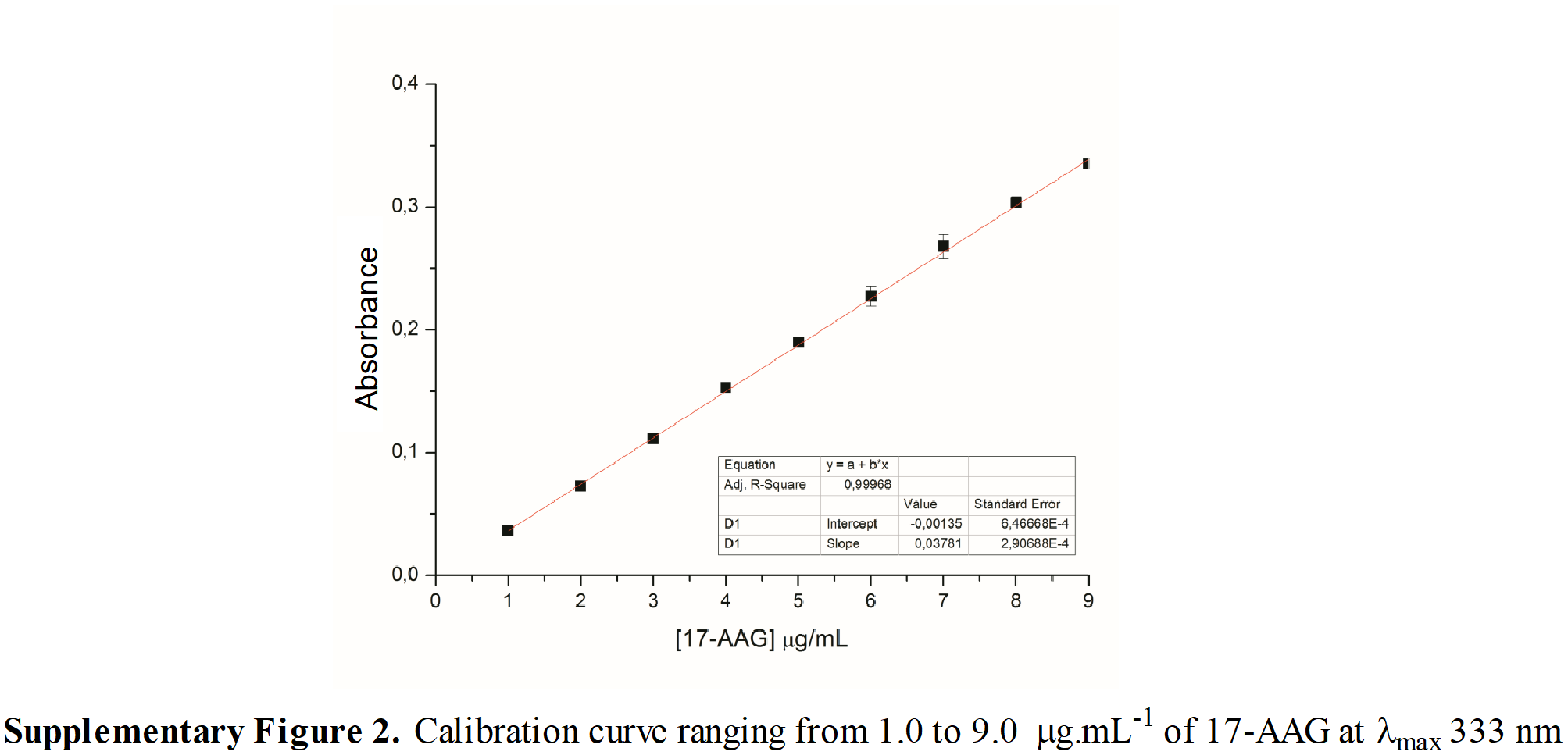

Supplement: Supplementary file 2 [file Image_2.TIF]

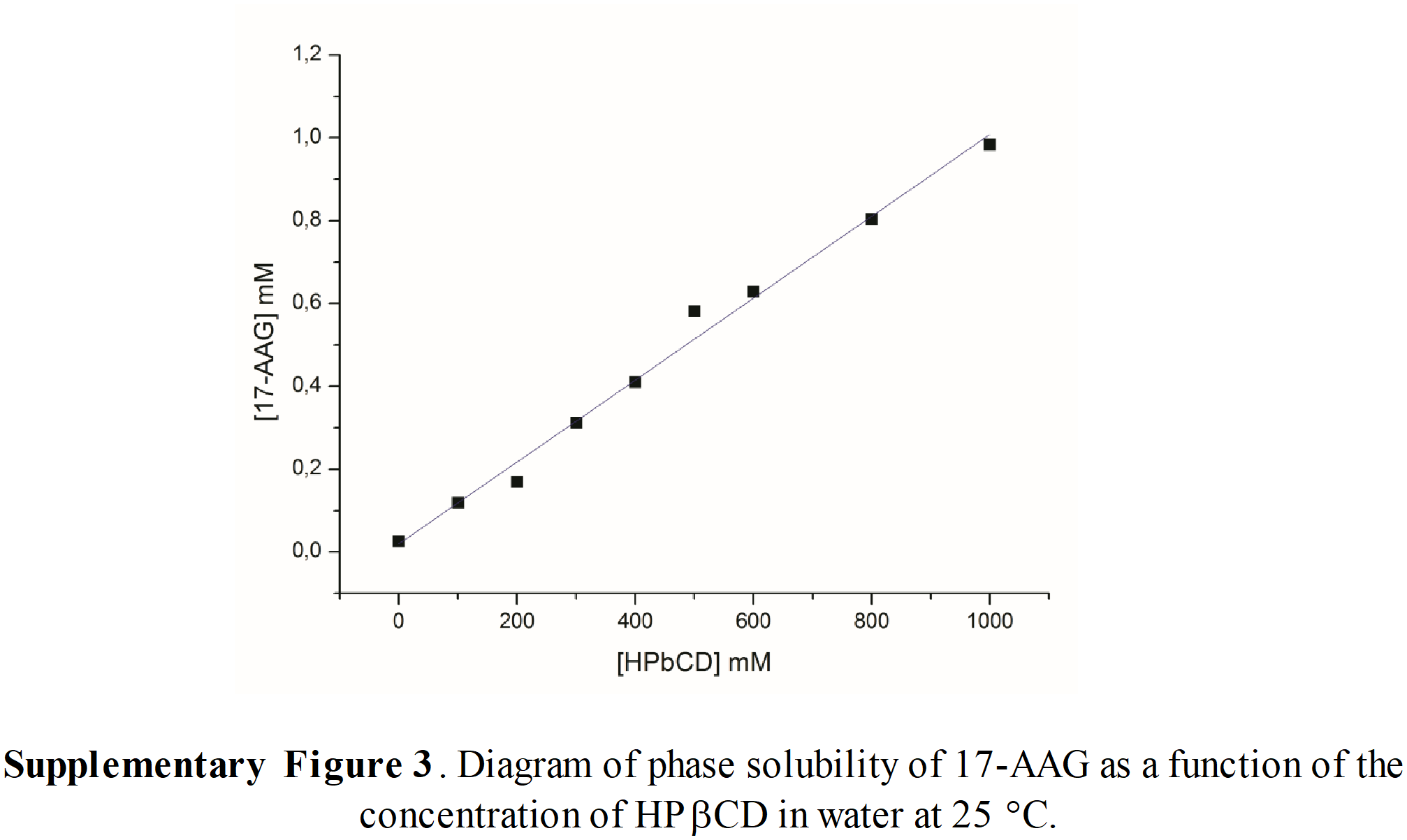

Supplement: Supplementary file 3 [file Image_3.TIF]

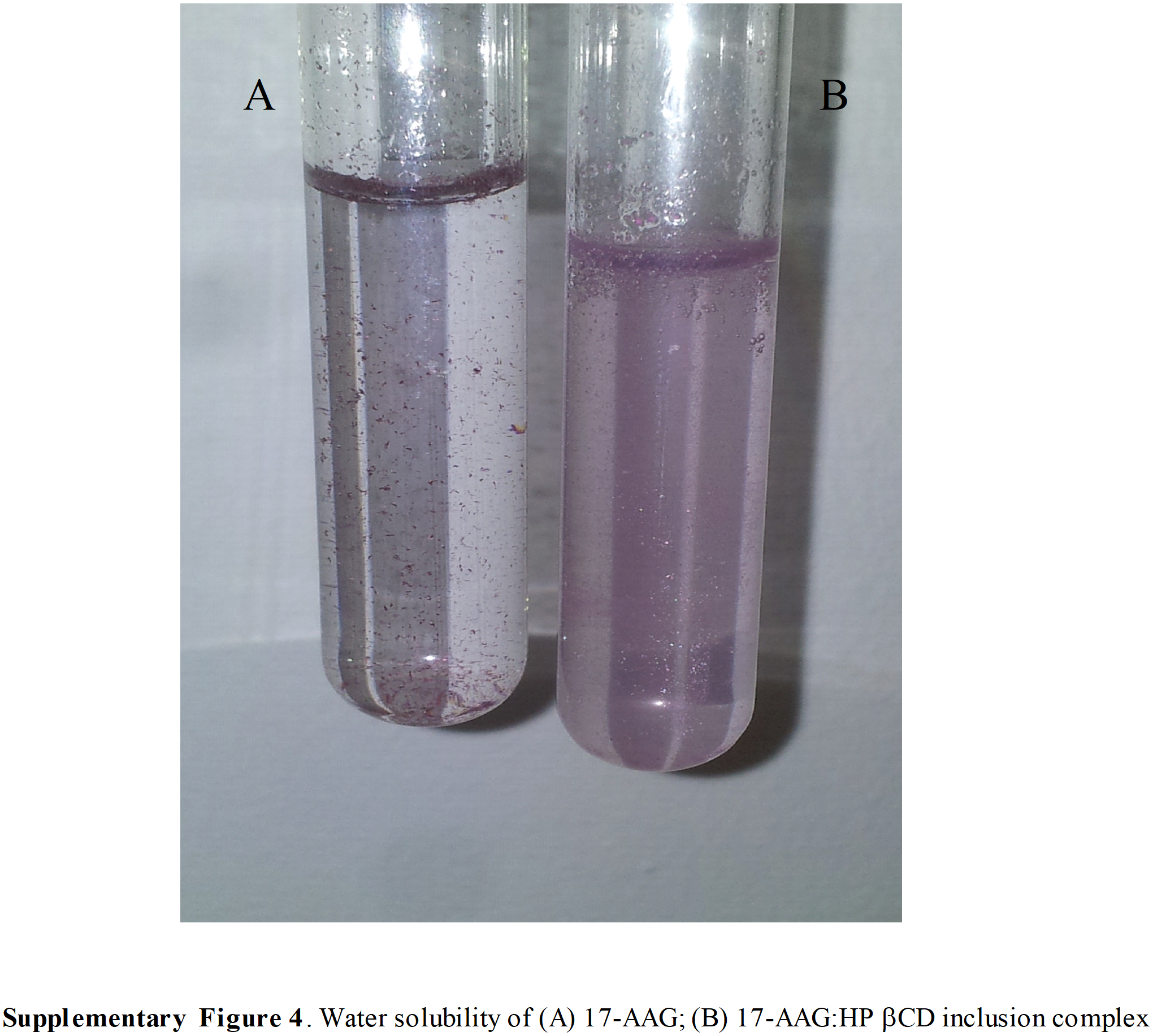

Supplement: Supplementary file 4 [file Image_4.TIF]

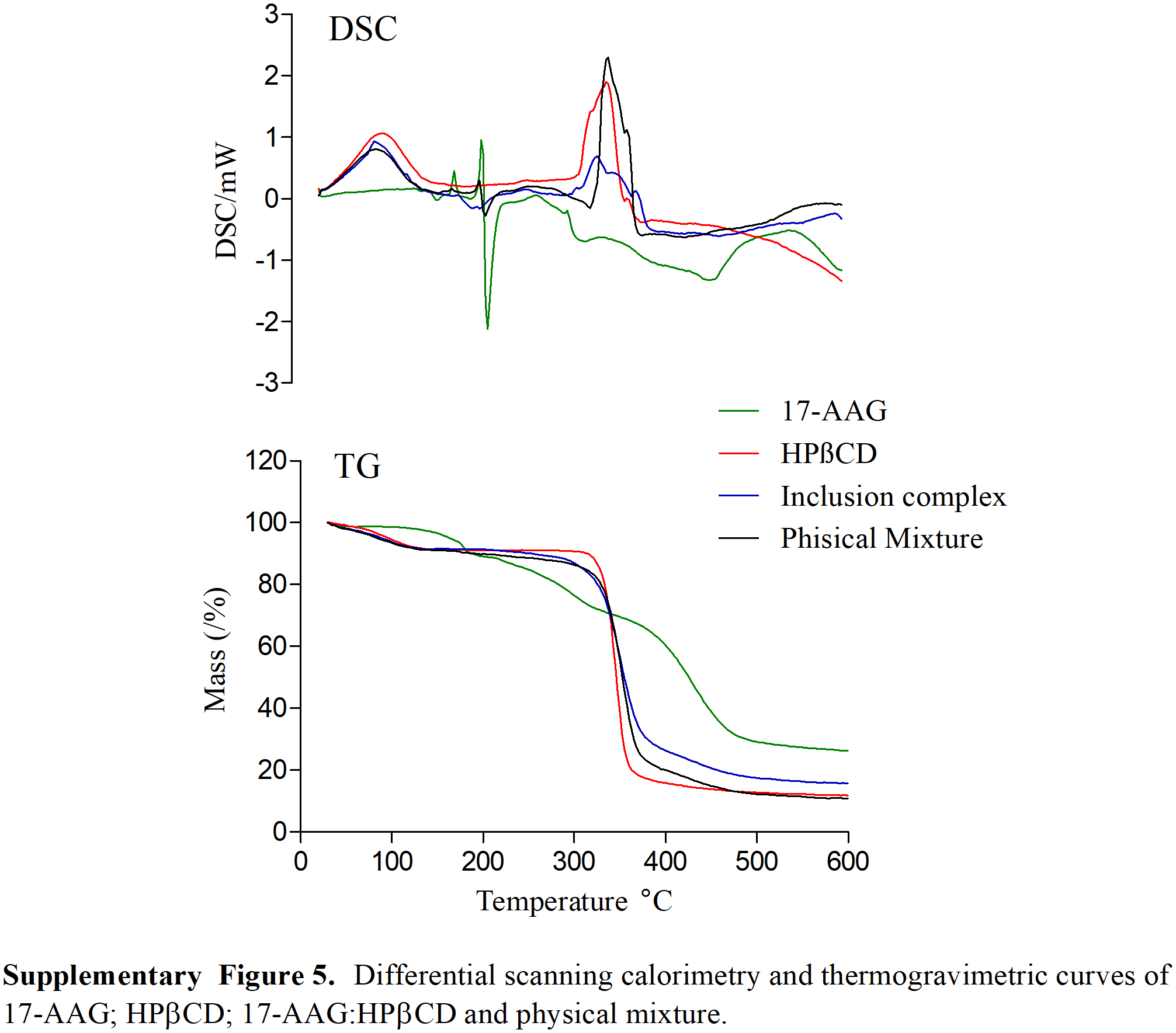

Supplement: Supplementary file 5 [file Image_5.TIF]
